# Experimental and numerical replication of blunt ballistic impact on a male thorax surrogate: study of rib fractures and lung injuries predictions

**DOI:** 10.3389/fbioe.2025.1704069

**Published:** 2025-12-04

**Authors:** E. Dancerel-Bourlon, R. Delille, B. Bourel, O. Mauzac, N. Prat, C. Bir, D. Sherman, S. Roth, F. Lauro

**Affiliations:** 1 Université Polytechnique Hauts-de-France, CNRS, UMR 8201 - LAMIH - Laboratoire d’Automatique de Mécanique et d’Informatique Industrielles et Humaines, Valenciennes, France; 2 French Ministry of the Interior, CREL/SAILMI, Paris, France; 3 Institut de Recherche Biomédicale des Armées (IRBA), Brétigny-sur-Orge, France; 4 Department of Biomedical Engineering, Wayne State University, Detroit, MI, United States; 5 Laboratoire Interdisciplinaire Carnot de Bourgogne, site UTBM, UMR 6303, CNRS /Université de Technologie de Belfort-Montbéliard, Belfort, France

**Keywords:** behind armour blunt trauma, thorax injuries, ballistics impacts, injury prediction, physical surrogate, numerical surrogate

## Abstract

Prediction of body injuries caused by non-penetrating ballistic impacts is a major challenge. Experiments on biological models are difficult to conduct due to ethical and logistical constraints and on the data that can be collected. These limitations can be overcome by using surrogates, but their results must be correlated with real injuries recorded by ballistic injury databases. The objective of this study is to establish injury prediction curves for rib fractures and lung injuries. Several ballistic impact scenarios from French and American police reports were analyzed. These cases were recreated experimentally using a biofidelic thoracic surrogate, SurHUByx, and numerically using its numerical twin, SurHUByx FEM. These surrogates represent the thorax of a 50th percentile male and were designed to reproduce the biomechanical responses of the human body to ballistic impacts. Then a scaling process adjusted the data according to body mass index to account for individual variability. The optimal cutoff for injury diagnosis was determined using the Youden method. And the injury risk curves were constructed using survival analysis according to ISO guidelines. The results show the risk of injuries according to rib deformation and pulmonary pressure over time. The injury probability was also plotted against the backface deformation. For a standard 44 mm intrusion, the risk of rib fracture was 44.84% and lung injury was 52.45%. These results highlight the limitations of current body armour standards, which may underestimate the probability of internal injuries. This study highlights the value of sharing ballistic injury cases to build a more reliable database. It also highlights the use of surrogates to replicate cases in order to develop injury prediction models.

## 1 Introduction

Law enforcement and military personnel are exposed to the threat of ballistic injuries in occupational situations (Bass et al., 2006). To reduce these risks, personal protective equipment such as bulletproof vests are used. However, these do not guarantee absolute protection against injury. Even in the absence of projectile penetration through the armour, ballistic impacts can still cause severe injuries, called Behind Armour Blunt Trauma (BABT). These injuries result from the interaction between impact energy and dynamic deformation at the back of the bulletproof vest (McMahon et al., 2025).

Currently, Roma Plastilina No. 1, an oil-based modeling clay, are the standard material for the evaluation of body armour. The armour is placed on the clay and impacted with test projectiles specified by the standard. Several impacts are made per panel. The acceptability of the results is determined based on one of two conditions: either each individual backface deformation (BFD) measurement in this material not exceed 44 mm, or one individual BFD values fall between 44 and 50 mm, then the average of all recorded impact measurements for a sample of the same model remains below 44 mm. Any measurement exceeding 50 mm is considered a failure (NIJ, 2023).

The 44 mm limit corresponds to the dynamic cone amplitude recorded in 20% ballistic gelatin under identical experimental conditions of ballistic aggression and protection as those applied in tests on goats, for which no mortality was observed (Goldfarb et al., 1975). Roma Plastilina No. 1 was chosen to simplify the test method. The measured cavity depth was equivalent to the dynamic cone amplitude in 20% ballistic gelatin. However, the static deformation of Roma Plastilina No. 1 and the dynamic deformation of 20% ballistic gelatin were found to be greater than the dynamic deformation of the goat thorax (Prather et al., 1977).

Other materials are commonly used in ballistic impact replication to mimic human tissue, such as 10 or 20% ballistic gelatin, Perma-gel and ballistic soap (Read et al., 2022). Styrene-Ethylene-Butylene-Styrene (SEBS) blocks are also commonly used to assess the effectiveness of ballistic protection (Bracq et al., 2021; Gilson et al., 2020). The advantage of this type of material is that it can track the deformation of the back of the panel over time.

However, human anthropometry must also be taken into account. The human thorax is not a homogeneous structure, since it is composed of hard and soft tissues and organs. Each region of the body reacts differently to impacts and has different injury tolerance thresholds (NIJ, 2023; Dos Santos et al., 2025). This requires specific studies for each area and biological structures of interest. A single threshold of protection does not offer the same level of protection for all skeletal structures and organs. Regional injury tolerances are needed to improve the safety of law enforcement and military personnel.

Modeling injuries caused by non-penetrating ballistic impacts is a major challenge (Chaufer et al., 2023; Dos Santos et al., 2025). Four approaches exist to better characterize the injury mechanisms associated with blunt impacts.

The first two are based on direct experimentation, which are classic methods for assessing ballistic injuries (McMahon et al., 2025; Prat et al., 2012). On the one hand, experiments on animals, although limited by anatomical differences with humans, are used. On the other hand, experiments on post-mortem human subjects (PMHS), which, despite their biomechanical relevance, have ethical and logistical constraints that severely limit these experiments.

The other two approaches focus on the use of anthropomorphic numerical and physical models. Numerous numerical models have been developed, such as HUByx (Bracq et al., 2019; Roth et al., 2013), SurHUByxFEM (Chaufer et al., 2023), SHTIM (Nsiampa, 2011), and WALT (Cronin et al., 2021). These numerical surrogates help understand complex phenomena that cannot be analyzed experimentally. For physical models, only a few surrogates have been developed, such as BTTR (Bolduc and Anctil, 2010) and the hybrid physical-numerical models developed “Gelman” (Roberts JC. et al., 2007; Roberts J. C. et al., 2007) and “SurHUByx” (Chaufer et al., 2024). However, the effectiveness of these approaches, numerical or physical, relies on their ability to faithfully reproduce human biomechanical responses to enable the replication and analysis of ballistic impacts.

In this study, the SurHUByx surrogate and its finite element version, SurHUByx FEM, were used. These surrogates were specifically developed to simulate a 50th percentile male thorax in the context of ballistic impact, and are considered numerical and physical twin models facilitating the recreation and analysis of ballistic impacts.

In ballistics research, collecting BABT cases remains a major challenge. Several studies have already reported case reconstructions in the literature (Bir et al., 2017; Chaufer et al., 2023; Sherman et al., 2023). To replicate cases on surrogates under conditions identical to those described in the case reports, various parameters must be known. Then, the values ​​measured on the surrogates are combined with injury data from the reports to determine whether an injury has occurred.

The main objectives of this paper are (1) to establish a database of BABT real life cases for replication, (2) to identify the most relevant experimental and numerical data suitable for defining injury criteria, and (3) to develop injury risk probability curves consistent with ISO standards. Additionally, the study aims (i) to give a methodology for replicating real cases through both experimental and numerical surrogates, and (ii) to introduce a normalization approach for data according the real cases anthropology.

## 2 Materials and methods

The following section outlines the ballistic recreation cases forming the basis of this study, the experimental and numerical methods employed, the data processing procedures, and the overall experimental evaluation approach. In the experimental setting, identical bulletproof vests and projectiles were used to ensure the conformity of the bulletproof vest model with the reported data. In the numerical setting, a process was performed to identify the overall behavior of the bulletproof vest and the projectiles. Data from the physical surrogate sensors and data from the numerical surrogate were scaled based on the body mass index (BMI) defined in the case reports. The sensor values ​​were scaled to account for variations between individuals described in the case reports. Then, injury probability curves corresponding to a 50th percentile male were generated following the ISO approach (2013). The data will then be compared to the NIJ standard (2023), as in the (McMahon et al., 2025). However, the relationship between the backface intrusion in the Roma Plastilina No. 1 (44 mm) and the physiological response of the human thorax remains insufficiently understood (Sarhan et al., 2023).

### 2.1 Case reports

**FIGURE 1:**
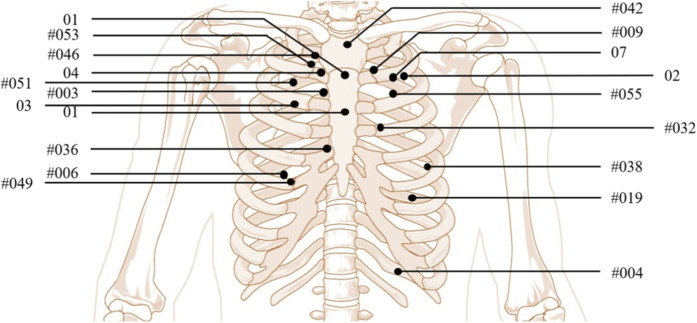
Location of impacts relative to the ribs.

In this study, ballistic impact scenarios described in French and American police BABT injury reports were analyzed. The objective was to gather relevant data to reproduce these situations under experimental and numerical conditions. Key information included physical subject characteristics, the type and model of bullet-resistant vest worn, precise impact locations, injury descriptions, weapons, and projectiles, including their velocities. In order to reduce variability between the cases studied, the analysis focused on frontal impacts involving the thoracic region of male subjects. Given the difficulty of grouping these cases, the body size of the individuals described was not an exclusion criterion. The collected data were organized into Table 1, which summarizes the selected cases, including subject characteristics and identified injury types. The injuries mentioned correspond to those described in the case reports. For the ribs, this corresponded to rib fractures. For the lungs, this corresponded to pulmonary contusions. The severity of the trauma was not taken into account, as the case reports do not provide data on the volume of contusions. In each case, an injury value was assigned as 0 when no injury and one when an injury was present. This method was systematically applied to all cases, to create a homogeneous dataset for analysis. Additionally, Figure 1 illustrates the location of bullet impacts relative to the ribs, as mentioned in different medical records. Each number corresponds to the case detailed in Table 1. In this study, 18 cases were replicated under experimental conditions and 15 cases under numerical conditions.

**TABLE 1:** Characteristics of individuals and their lesions.

Case references		Case reports type	Subject characteristics		Projectile	Impact velocity [m/s]	Body armour		Rib fractures	Lung lesion	Additional information	Recreation
			Height [m]	Weight [kg]								
BABTID #003 (Bir et al., 2017)		US case	1.73	72.5	Lawman 9 mm 124 gr FMJ (Full Metal Jacket)	315	Second Chance	Monarch 329-MON-II	0	0	-	Exp. only
BABTID #004 (Sherman et al., 2023)		US case	-	-	Remington 0.40 calibre S&W 180 gr	301	Second Chance	Ultima SMU-II + 001,221	1	0	9th rib fracture	Both
BABTID #006 (Bir et al., 2017)		US case	1.75	74.5	Lawman 9 mm 124 gr FMJ	356	Second Chance	MON-II + LSC	0	0	-	Both
BABTID #009 (Sherman et al., 2023)		US case	1.80	88.45	RWS 0.38 special 158 gr FMJ	263	PACA	KSG IIIA	0	0	-	Both
BABTID #019 (Bir et al., 2017)		US case	1,75	75	Speer 9 mm 115 gr HP (Hollow Point)	366	ABA	XT2-1	1	0	8th rib fracture	Both
BABTID #032 (Cronin et al., 2021)		US case	1.85	113.4	0.38 calibre	296	ABA	XT3A-2	0	0	-	Both
BABTID #036 (Bir et al., 2017)		US case	1.83	100	Winchester 0.45 calibre, 230 gr, FMJ	250	Safariland	GX-II.0	0	0	-	Both
BABTID #038 (Sherman et al., 2023)		US case	1.70	63.5	0.38 calibre, FMJ	259	Safariland	SII-6.0	0	1	Underlying pulmonary contusion in anterior left lower lobe	Exp. only
BABTID #042 (Bir et al., 2017)		US case	1.65	64	Federal Premium 0.40 calibre S&W 180 gr HP	305	Point Blank	CIIA-1	0	1	Bruised lungs	Both
BABTID #046 (Bir et al., 2017)		US case	1.78	75	Glock 0.45 230 gr JHP	241	Monarch	MON-11 107,121	0	1	Severe bruising and contusion	Num. only
BABTID #049 (Bir et al., 2017)		US case	1.90	100	Winchester 0.45 calibre 230 gr JHP	243	ABA	XT3A-2	0	0	-	Both
BABTID #051 (Bir et al., 2017)		US case	1.87	131.5	12 gauge	406	Monarch	SUM IIA R02 6010	0	1	Pulmonary contusion	Both
BABTID #053 (Sherman et al., 2023)		US case	1.88	70.31	Lawman 9 mm 124 gr TMJ (Total Metal Jacket)	322	Safariland	BA-2000S-SM01	0	1	Pulmonary contusion	Both
BABTID #055 (Sherman et al., 2023)		US case	1.85	68.04	Winchester 0.357 mag JHP	374	Safariland	BR01	1	0	2nd and 4th rib fracture	Both
01	Internal record of the French Interior Ministry	PMHS case	1.74	72	calibre 12 Brenneke	385	CDX 2 × 10 layers		1	-	6 rib fractures	Both
02		PMHS case	1.77	50	calibre 12 Brenneke	435	CDX 2 × 10 layers		1	-	1 rib fracture	Exp. only
03		PMHS case	1.65	52	Gévelot 9 mm Para. 8.18 g	378	CDX 10 layers		0	0	-	Exp. only
04		PMHS case	1.60	47	Gévelot 9 mm Para. 8.18 g	370	CDX 20 layers		0	0	-	Both
07		French case	1.76	80	0.38 calibre special JHP	250	COMODITEX GN 2001		0	0	-	Both

### 2.2 Anthropomorphic surrogate

This study aims to recreate BABT cases using both experimental and numerical approaches. Surrogates, previously designed and validated, which allows for the development of injury risk curves. In this context, the SurHUByx FEM surrogate and its physical twin were used. This will allow for a comparison of experimental and numerical results.

#### 2.2.1 The numerical twin: SurHUByx FEM

**FIGURE 2:**
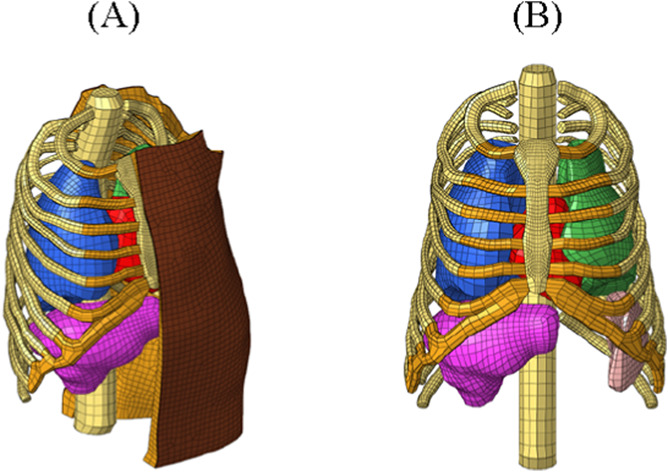
SurHUByx FEM **(A)** and SurHUByx FEM without muscle and skin **(B)**.

The SurHUByx FEM model is a simplified finite element (FE) version of the biofidelic thoracic model HUByx (Roth et al., 2013), representing a 50th percentile human thorax. SurHUByx FEM was designed to have a manufacturable structure (Figure 2A). To achieve this, SurHUByx FEM relies on a geometry optimized for manufacturing (Chaufer et al., 2023).

Several simplifications were made. Some anatomical structures were merged. The thoracic cage structure was simplified, with homogenization of the mechanical properties of bones, cartilage, and trabecular tissue (Figure 2B). The vertebrae were merged.

The initial materials of the HUByx model were replaced by material laws based on available materials with mechanical properties close to those of human tissues.

SurHUByx FEM, developed with Hypermesh and solved with Radioss. It consistently reproduces the biomechanical response observed with experimental data from cadaveric tests under ballistic impact conditions. (Chaufer et al., 2024; 2023). SurHUByx FEM is part of an approach to developing numerical models compatible with industrial manufacturing processes, while contributing to the design of physical twins dedicated to ballistic applications.

#### 2.2.2 The physical twin: SurHUByx

**FIGURE 3:**
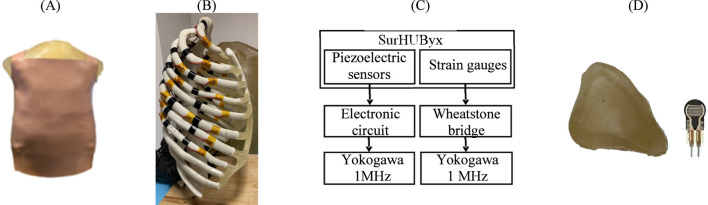
SurHUByx **(A)** Strain gauges on the ribs **(B)** SurHUByx data acquisition chain **(C)** and piezoelectric sensors used in internal organs **(D)**.

The SurHUByx geometry (Figure 3A) was developed from the mesh used in the SurHUByx FEM model, which represents a 50th percentile male thorax. The surfaces were imported into CATIA V5 design software. SurHUByx is composed of several types of materials to model the different components. Finally, SurHUByx was equipped with sensors positioned on the ribs and in the lungs to measure deformations and pressures during ballistic impacts (Chaufer et al., 2023).

To predict rib injuries, strain gauges were installed on the ribs (Figure 3B). These gauges had a sensitivity of 3% with a resistance of 350 Ω. Data were recorded using a Yokogawa DL750 recording oscilloscope with a sampling rate of 1 MHz. Ribs one to nine were equipped with one–two gauges each, resulting in approximately 30 gauges distributed across all the ribs of the surrogate. Due to the limited number of ports available on the YOKOGAWA, only 15 gauges could be connected simultaneously. Therefore, the 15 strain gauges near the impact zone were connected to the oscilloscope (Chaufer et al., 2023).

In addition, piezoelectric pressure sensors, placed at the centre of each internal organ, measured the pressure generated during impacts (Figure 3D). These sensors require an electronic circuit, shown in Figure 3C. A circuit board, powered by a DC generator, is specially designed to integrate the electronic circuit into the assembly and facilitate the connection of the sensors during testing. These uniaxial sensors, whose activation sensitivity is approximately 0.2 N, required frontal shots to ensure the reliability of the measurements. The signals were also recorded via the Yokogawa DL750 oscilloscope, at a sampling rate of 1 MHz.

#### 2.2.3 Validation of SurHUByx FEM and SurHUByx in the ballistic field

The SurHUByx FEM surrogate, along with its physical twin, were employed in this study to simulate ballistic impacts on the thoracic region. Specifically designed to replicate the biomechanical response of a 50th percentile male thorax subjected to ballistic impact, these models were validated by comparison with experimental data reported by Bir et al. (2004).

The reconstruction of PMHS tests enabled the comparison of displacement-time and force-time curves with the biomechanical corridors from the literature (Bir et al., 2004). These comparisons highlighted good agreement between the numerical data and experimental data. The overall consistency of the responses, both numerical and physical, with the corridors validates the reliability of the models in the context of reproducing ballistic impacts (Chaufer et al., 2024).

### 2.3 Experimental replications of case reports

#### 2.3.1 Extract data

**FIGURE 4:**
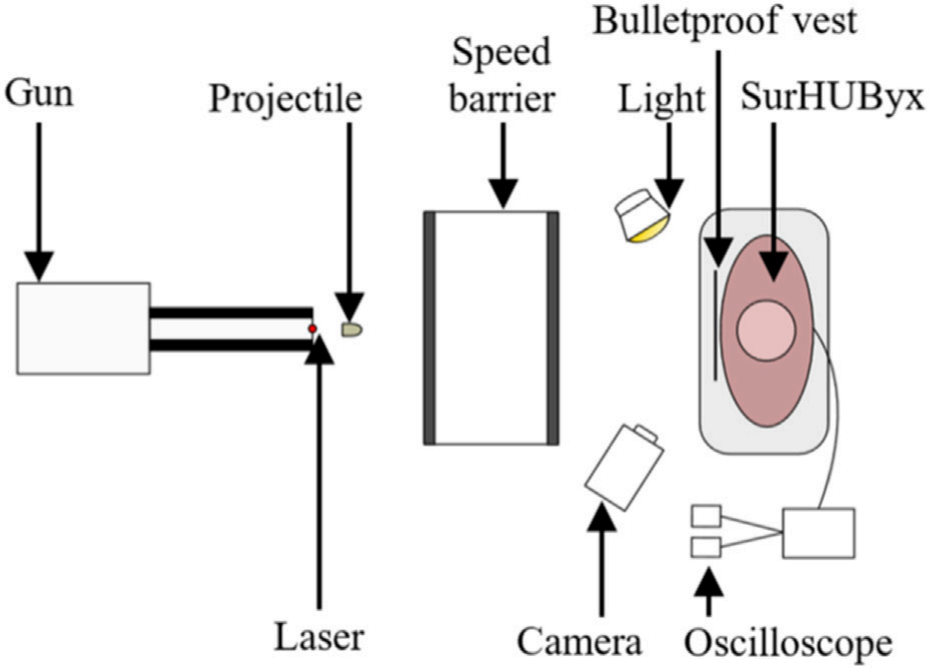
The setup of the experimental device.

Figure 4 shows the experimental setup used to recreate ballistic impacts on the SurHUByx physical surrogate. SurHUByx was positioned 7.5 m from the gun. Before each impact, a calibration shot was performed to ensure that the projectile was launched at the desired velocity to replicate the specific case. After aligning the intended point of impact using a laser sight, the projectile was fired at the surrogate. The projectile velocity was recorded using an IR velocity gate. The impact on the ballistic vest was captured by a high-speed camera (Phantom v2012) at a rate 22,626 frames per second. To recreate a case, the same type of projectile specified in the case reports was used, along with bulletproof vest models manufactured within 5 years of the one worn by the officer. Due to the limited surface area on bulletproof vests, each case was only reproduced once on SurHUByx. A complete assessment of the surrogate was conducted between each impact to ensure its physical integrity. If any damage was detected on the SurHUByx surrogate, the necessary repairs were carried out before continuing the experimental tests. For example, repairs could involve replacing the sternum or cartilage substitute if broken or reinstalling a strain gauge.

#### 2.3.2 Data analysis

For each studied case, only the maximum value of the sensor considered recorded for each organ was retained, representing a single measurement per internal organ.

Regarding strain gauges, to quantify injuries, the maximum value recorded by the strain gauges throughout the test was selected. This value was extracted directly from the raw data.

The same principle applies to the piezoelectric pressure sensors, to quantify lung injury, the maximum value recorded by the piezoelectric sensors inside both lungs throughout the test was selected. This value was extracted directly from the raw data.

### 2.4 Numerical replications of case reports

#### 2.4.1 Define the equivalent parameters of the projectile and the bulletproof vest

**FIGURE 5:**
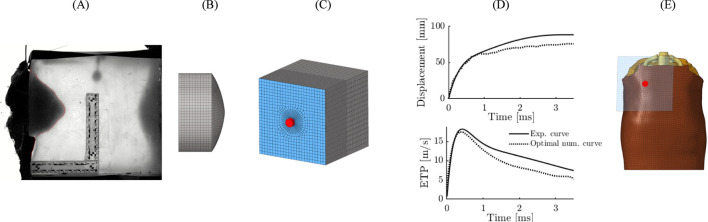
Reverse engineering approach applied for numerical replication. BFD on gel block **(A)** modeling of the projectile **(B)** Modeling of the experiment on gel block **(C)** Result of the optimization of the parameters **(D)** Modeling of the case report **(E)**.

Replicating the impact case on a gel block allows, through video analysis, measuring the temporal evolution of the experimental BFD (Figure 5A). It also allows knowing the properties of the projectile after impact: mass, diameter, and length. From this deformed geometry, an equivalent projectile was reconstructed for numerical replication. This projectile was discretized into brick-type finite elements and was considered rigid (Figure 5B). As for the bulletproof vest, it was modeled with a single layer of shell elements, and an anisotropic hyperelastic law (M58_FABR_A) was used in the explicit Radioss solver. An initial numerical simulation was then performed (Figure 5C), and the evolution of the BFD was measured and compared with the experimental results. Next, the parameters of the bulletproof vest and the equivalent projectile were optimized using HyperStudy software, aiming to minimize the discrepancies between numerical simulations and experimental tests. The parameters modified during the optimization were the density, shear modulus, and thickness of the vest, as well as the initial back face velocity. When the difference in the projectile trajectory between the numerical and experimental results was less than 20%, the identified parameters were extracted (Figure 5D). Then, during the numerical replication of the case, the optimal data of the projectile and the bulletproof vest were imported into the SurHUByx FEM numerical model. Since the model parameter optimization was performed for a flat surface, it was decided not to modify the vest geometry or its mesh (Figure 5E).

#### 2.4.2 Replication on SEBS gel block

**FIGURE 6:**
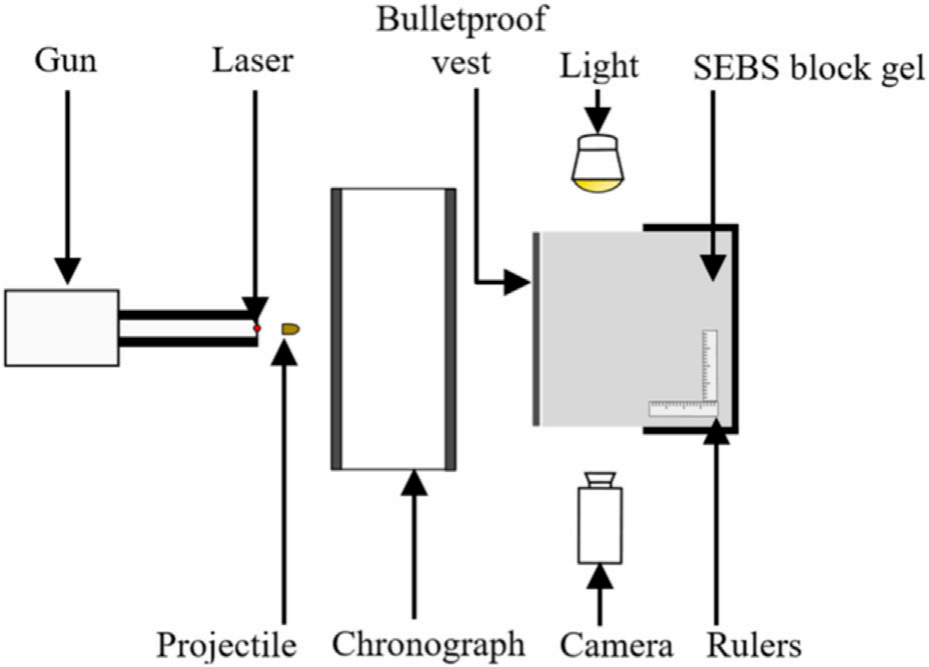
The experimental setup for SEBS gel block tests.

To obtain the parameters of the mechanical behaviour of the vest a procedure was applied based on an experimental test on a system: gel block/body armour. These impacts were numerically reproduced to determine the overall parameters of the vest using an inverse method. Indeed, the objective here is to model the overall behavior of the gel block/body armour system. The precise parameters of the vest are not relevant to this study. Figure 6 illustrates the experimental setup used to recreate ballistic impacts on the SEBS gel block, for the purpose of measuring BFD. The gel block was positioned at a distance of 7.5 m from the weapon. Before each impact, a calibration shot was taken to ensure that the projectile velocity matched that specified in the case report. The projectile velocity was recorded using an infrared velocity gate. The impact on the body armour was captured by a high-speed camera (Phantom v2012) at a rate of 25,000 frames per second. The body armour and projectiles conformed to the description in the case reports. Due to the limited surface area on bulletproof vests, each case was only reproduced once on gel block.

Cases BABTID #004, #009, #019, #042, #046, and #049 were replicated in a previous study. Given that the experimental methodology employed was identical, the results obtained in this previous study (Bracq, 2018) were incorporated into the current analysis.

The methodology described above was applied to all case reports. After numerical reproduction of each case, different variables were analyzed to identify those with the best predictive capacity for ribs and lungs.

Note that, the aim of this work was not to compare or correlate the backface deformation results obtained with the gel block and the physical/numerical surrogate. Replicating the case on the gel block only allowed to define the equivalent properties of the projectile and the bulletproof vest as defined in Bracq (2018). The backface deformations are different between the gel block and the surrogate as the backing are different but the results properties of the projectile and the bulletproof vest are still the same.

#### 2.4.3 Extract data

For the numerical surrogate, an analysis was conducted on several variables based on the organs involved. The variables observed were von Mises stresses, von Mises strains, normal strain along the X-axis in the local frame, specific energy, skin deflection at the point of impact, as well as pressure and the pressure/time curve at the lungs. Two methods were compared for data extraction. The first consists of retaining only the maximum value obtained during the simulation. The second considers the average between this maximum value and those of its four neighbouring points. The latter method proved more relevant for interpreting the results. The choice of variables was guided by current normative recommendations. Among all the variables, the most relevant in this case study to distinguish the injury cases from the no-injury cases were the skin deflection at the point of impact, the normal strains along the X-axis at the ribs, as well as the pressure/time at the lungs.

### 2.5 Data analysis

Now that the protocol used to experimentally and numerically recreate the reported impacts is described, the method used to scale the data and create the injury risk probability curves can be presented.

#### 2.5.1 Data scaling

In the case reports presented in Table 1, individuals had different physical characteristics in terms of mass and stature. These differences made the interpretation of the data more difficult. Given that, individual variations in these parameters influence the response of body structures to ballistic forces (Robbe, 2013), a scaling approach was implemented on the values ​​obtained by the SurHUByx surrogate and the SurHUByx FEM surrogate. Thus, the results of each replicated case were adjusted according to the BMI of a 50th percentile male, with reference values, 77 kg for mass and 1.78 m for stature, as explained by (Serre et al., 2006). BMI is used to assess the body composition of an individual, as defined by:

$$\text{BMI}=\frac{\text{Mass}\;\left[\text{kg}\right]}{{\text{Stature}}^{2}\left[m^{2}\right]}$$

This approach made it possible to calculate a specific scale factor for each individual (Eppinger et al., 1984):

$$\lambda =\frac{{\text{BMI}}_{50\text{th}}}{{\text{BMI}}_{\text{subject}}}$$

Once the factor was obtained, it was applied to the data collected by the physical and numerical surrogates, multiplying the data by the corresponding scaling factor. The results are thus scaled, facilitating their interpretation. This operation normalizes the results, thus facilitating their comparison and interpretation.

For the physical surrogate, the scaled data correspond to measurements recorded by piezoelectric sensors placed in the lungs and by strain gauges attached to the ribs.

For the numerical surrogate, all variables extracted from the numerical simulations were scaled using the factor specific to each case studied.

#### 2.5.2 Creation of injury probability curves

The data were analyzed using survival analysis to assess the relationship between the probability of injury occurrence and the value of an independent variable representing a mechanical parameter. The data obtained from the surrogate subject were then compared to the injuries reported in the reported cases (Table 1). In this study, the analysis was performed using MATLAB R2024b. The proposed approach was applied to establish risk curves for rib and lung BABT resulting from ballistic impacts on the thorax. The International Organization for Standardization (ISO) has developed a step-by-step process for developing injury risk functions (ISO, 2013), providing a foundation for the use of a unified approach to constructing injury risk curves. This standard has been used in several previous studies (McMahon et al., 2025; Yoganandan et al., 2016).

The steps of the ISO approach are as follows: (1) data collection, (2) determination of censoring status, (3) verification of multiple injury mechanisms, (4) sample separation based on injury mechanisms, (5) estimation of distribution parameters, (6) identification of highly influential observations, (7) verification of the distribution hypothesis, (8) selection of the appropriate distribution, (9) validation of predictions against existing results, (10) calculation of 95% confidence interval, (11) evaluation of the quality index, and (12) recommendation of a curve per body region (ISO, 2013).

The 12 steps of the ISO procedure provide a structured approach, but they do not address all the nuances of the problem. Subsequent research has refined this process to address existing gaps (Robbe, 2013). The ISO approach did not provide a method for determining which biomechanical measure from the experiments was most correlated with injury outcomes. To address this, a process of selecting the measure with the highest area under the curve (AUC) was proposed (Yoganandan et al., 2016). The Receiver Operating Characteristic (ROC) curve evaluates the discriminative ability of biomechanical variables the concerning injury outcomes. It is obtained by plotting sensitivity against 1-specificity for different classification thresholds. The AUC represents the predictability measure of metric in terms of specificity and sensitivity. A higher AUC indicates a more predictive measure. The AUC was calculated for all potential criteria, and the metric with the highest AUC was selected for further analysis.

Once the most relevant criterion is identified, a survival analysis was performed to estimate the parameters associated with the different distributions (ISO). In this study, the Kaplan-Meier curve was plotted, a nonparametric method used to estimate the survival function. The inverse survival curve was analyzed to illustrate the cumulative probability of injury. To complement the evaluation of the model, an influence analysis of the data was conducted using the DFbeta statistic (Belsley et al., 2005). DFbeta measures the impact of each observation on the survival model coefficients. A high DFbeta value indicates a potentially influential observation.

Then, the studied parametric survival models were those recommended by the ISO standard. The fitted models included Weibull, log-normal, and log-logistic distributions. For each distribution, the Akaike Information Criterion (AIC) was computed to select the most suitable model:

$$\text{AIC}=-2\times \log\left(L\right)+2\times k$$

In the formula, L represents the maximized likelihood of the model, which measures how well the model fits the data. The term k represents the number of parameters in the model, including the intercept and any additional predictor.

Confidence intervals were then computed to quantify estimation uncertainty. The confidence interval 95% estimates a range in which the true parameter value is expected to lie with a given probability.

In parallel, based on the obtained ROC curves, the Youden index was applied to determine the optimal threshold that maximizes the effectiveness of injury diagnostic testing. It is denoted as J:

J=Sensitivity+Specificity−1

A higher Youden index indicates a better diagnostic test. In this study, misclassification of a positive individual was considered more detrimental than that of a negative individual (Tasu et al., 2025).

## 3 Results

A total of 18 BABT impact tests were conducted on the SurHUByx surrogate and 15 BABT impact on the SurHUByx FEM, in the sternum and costal cartilage region. The measurements were scaled according to the BMI of the individuals. When individual characteristics were unknown, the case was excluded; in this study, therefore, case BABTID#004 was excluded.

### 3.1 Sensor signals

**FIGURE 7:**
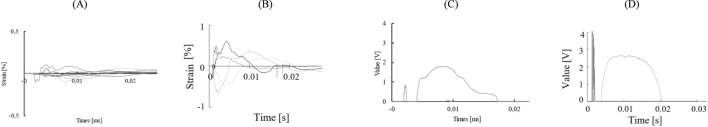
Curves obtained from strain gauges for BABTID#036 **(A)** and case 02 **(B)** and from piezoelectric pressure sensors for BABTID#036 **(C)** and BABTID#051 **(D)**.

When analyzing the sensor signals on the physical surrogate, the recordings show the presence of a “double peak” phenomenon in the time curves obtained from the piezoelectric pressure sensors (Figures 7C, D). The first peak was due to the shock wave caused by the initial impact. The second peak, which lasted longer, was due to the interaction between the bulletproof vest and the substitute. Therefore, the analysis focused on this second peak.

Moreover, when the replicated case did not involve any rib fractures, the recorded curves showed low amplitudes (Figure 7A). Conversely, when at least one rib fracture was reported, at least one gauge recorded a high amplitude (Figure 7B). The same principle applies to the piezoelectric pressure sensors. The larger the signal, the stronger the pressure (Figure 7D), while a weak signal corresponds to a reduced pressure (Figure 7C).

### 3.2 Rib injuries predictions

**FIGURE 8:**
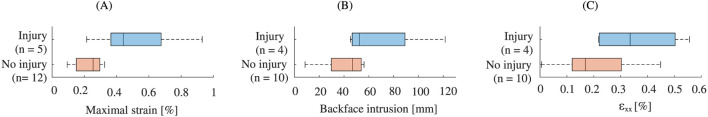
Boxplot of scaled rib data: Experimental **(A)** Numerical backface intrusion **(B)** and Numerical normal strain X values **(C)**.

Figure 8 illustrates the distribution of the experimental and numerical data on whether a rib injury was reported in the case. For the experimental replication data (Figure 8A), the “No Injury” samples have an average lower normal strain X value. It is 0.25% for the “No Injury” compared to 0.52% for the “Injury” samples.

For the numerical replication data, the “No Injury” samples also exhibit lower backface intrusion values (Figure 8B) on average. It is 42.06 mm compared to 67.81 mm for the “Injury” samples. For the variable normal strain X values (Figure 8C) on average, it is 0.20% for the “No Injury” compared to 0.36% for the “Injury” samples.

**FIGURE 9:**
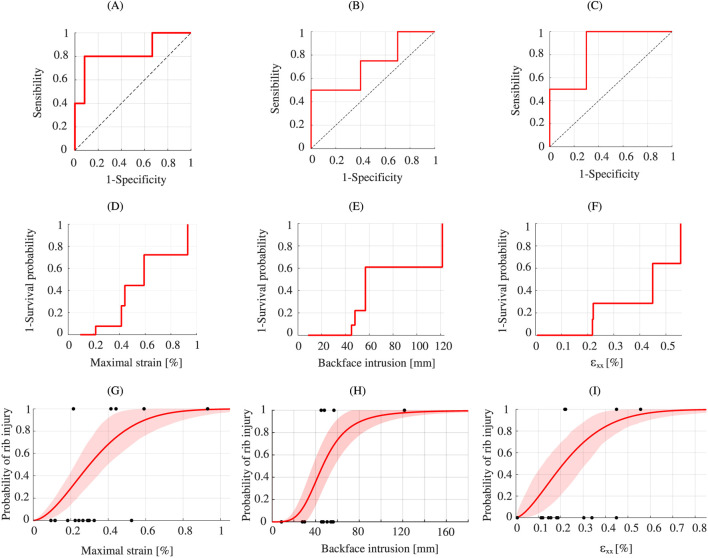
Ribs injury analysis. ROC curve of the ribs data: Experimental **(A)** Numerical backface intrusion **(B)** and Numerical normal strain X values **(C)**. Kaplan-Meier curve: Experimental **(D)** Numerical backface intrusion **(E)** and Numerical normal strain X values **(F)**. Best fit model for injury risk curves for ribs: Experimental - Weibull **(G)** Numerical backface intrusion - Log-logistic **(H)** and Numerical normal strain X - Weibull **(I)**.

The ROC curve of the analyzed variables was plotted. The experimental AUC for the normal strain X values is 0.833 (Figure 9A). The decision threshold was determined on the basis of the Youden index and the ROC curve. For the ribs, the threshold is 0.414% with a specificity of 0.917 and a sensitivity of 0.8 with J = 0.717.

The numerical AUC for the backface intrusion variable is 0.725 (Figure 9B). The decision threshold was determined on the basis of the Youden index and the ROC curve. The threshold is 47.97 mm, with a specificity of 0.6 and a sensitivity of 0.75 with J = 0.35. And for the normal strain X variable, the AUC is 0.85 (Figure 9C). The decision threshold was determined on the basis of the Youden index and the ROC curve. The threshold is 0.218%, with a specificity of 0.7 and a sensibility of 1 with J = 0.7.

The Matlab routine output the inverse Kaplan-Meier survival curve for experimental data (Figure 9D) and for numerical data (Figures 9E,F). Next, the three fitted models were plotted and analyzed. Compared with the AIC value obtained in the analysis (Table 2), the Weibull distribution is most appropriate in this study for experimental data. For the numerical data, the most appropriate distribution for backface intrusion is log-logistic. For the numerical normal strain X the most appropriate distribution is Weibull.

**TABLE 2:** Injury risk curve final predictor and distribution selections with test statistics.

Injury	Predictor	Distribution	AIC
Ribs injury	Experimental maximum strain with BMI scaling	Log-normal	33.2
		Log-logistic	26.3
		Weibull	**21.9**
	Numerical backface intrusion with BMI scaling	Log-normal	171.8
		Log-logistic	**150.0**
		Weibull	151.4
	Numerical normal strain X with BMI scaling	Log-normal	96.3
		Log-logistic	**27.4**
		Weibull	24.1
Lung injury	Numerical backface intrusion with BMI scaling	Log-normal	233.9
		Log-logistic	148.3
		Weibull	**141.4**
	Numerical pressure/time(peak-start) with BMI scaling	Log-normal	**17.4**
		Log-logistic	19.8
		Weibull	19.3

The Matlab routine generated the inverse Kaplan-Meier survival curve for the experimental (Figure 9D) and numerical (Figures 9E, F) data. The three fitted models were then plotted and analyzed. Compared with the AIC value obtained in the analysis (Table 2), the Weibull distribution is the most appropriate in this study for the experimental data.

The risk of rib injury following a frontal ballistic impact by experimental replication is highlighted in Figure 9G which illustrates the results of the rib injury risk curve with the Weibull distribution. For a risk of 50%, the maximum rib strain is 0.30% (±95% CI: 0.22%–0.39%).

Figure 9H highlights the risk of rib injury following a frontal ballistic impact using numerical replication for the backface intrusion. The results of the rib injury risk curve follow the log-logistic distribution. For a 50% risk, the backface intrusion is 46.38 mm. For the 44 mm body armour design criterion, the numerical surrogate produces a rib injury risk of 44.84% (±95% CI: 23.53%–66.53%).

Figure 9I illustrates the risk of rib injury from the numerical normal strain X of the ribs. The results of the rib injury risk curve follow the Weibull distribution. For a 50% risk, the deformation is 0.21% (±95% CI: 0.12%–0.31%).

### 3.3 Lung injuries predictions

**FIGURE 10:**
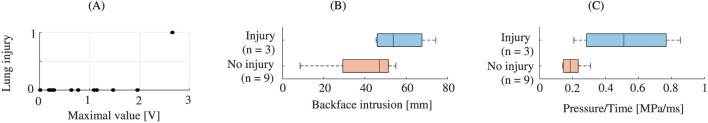
Boxplot of scaled lung data: Experimental **(A)** Numerical backface intrusion **(B)** and Numerical pressure/time **(C)**.

The sample size of the experimental data is unequal between the groups. Only one case represents the group “Injury” (Figure 10A). Indeed, including cases that occurred too far from the sensor would have introduced additional methodological biases. With only one experimental injury case in this study, generating risk curves for the lungs was not possible for the experimental lungs data.

Figure 10B illustrates the distribution of the numerical data on whether a lung injury was reported in the case. For the numerical replication data, the “No Injury” samples also exhibit lower backface intrusion values ​​on average. It is 40.62 mm compared to 56.82 mm for the “Injury” samples. And, with other variable (Figure 10C), the “No Injury” samples also exhibit lower pressure/time values ​​on average. It is 0.22 MPa/ms compared to 0.52 MPa/ms for the “Injury” sample.

**FIGURE 11:**
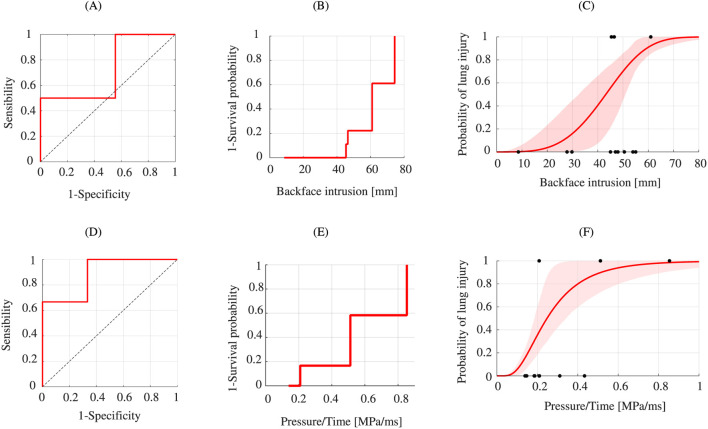
Lung injury analysis. ROC curve of the lung data: Numerical backface intrusion **(A)** and Numerical **(D)**. Kaplan-Meier curve: Numerical backface intrusion **(B)** and Numerical Pressure/Time **(E)**. Best fit model for injury risk curves for ribs: Numerical backface intrusion - Weibull **(C)** and Numerical Pressure/Time–Log-normal **(F)**.

The ROC curve was therefore plotted. The numerical AUC for the backface intrusion variable is 0.722 (Figure 11A). The decision threshold was determined on the basis of the Youden index and the ROC curve. For the lungs, the threshold is 38.06 mm, with a specificity of 0.4 and a sensitivity of 1 with J = 0.4. The numerical AUC for the pressure/time variable is 0.889 (Figure 11D). The decision threshold was determined on the basis of the Youden index and the ROC curve. The threshold is 0.208 MPa/ms, with a specificity of 0.667 and a sensitivity of 1 with J = 0.67.

Figure 11C highlights the risk of lung injury following a frontal ballistic impact using numerical replication. The results of the lung injury risk curve follow the log-logistic distribution. For a 50% risk, the backface intrusion is 43.19 mm. For the 44 mm body armour design criterion, the numerical surrogate produces a lung injury risk of 52.45% (±95% CI: 18.06%–74.21%). And, for the other variable (Figure 11D), Pressure/Time, for a 50% risk, the value is 0.248 MPa/ms (±95% CI: 0.186–0.342 MPa/ms).

## 4 Discussion

One of the strengths of this study is the use of a physical thorax surrogate, SurHUByx, and its numerical twin, SurHUByx FEM, both representing a 50th percentile male thorax. These surrogates couple allow for the estimation of the risk of certain BABT injuries, such as rib fractures and lung injuries. These approaches represent an ethical and reproducible alternative to testing on human or animal subjects (Chaufer et al., 2023).

Firstly, experimental tests were conducted using the physical surrogate. Pressure sensors and strain gauges installed in the surrogate allowed to the collection of experimental data to measure the mechanical deformations experienced on the thoracic structure of the SurHUByx model. The recordings (Figure 7) highlight the presence of a “double peak” phenomenon on the time curves obtained from piezoelectric pressure. Similar observations were reported (Han et al., 2020; Luo et al., 2016) for a ballistic impact on a gelatin block protected by a bulletproof vest. The first peak was due to the shock wave caused by the initial impact between the projectile and the body armour, characterized by a sudden initial pressure peak of short duration. The second peak, longer in time, was due to the interaction between the deformed body armour and the gelatin block (Bass et al., 2006). Therefore, the analysis focused specifically on this second peak. These data were then used to estimate the probability of lesions by BABT.

The correlation between biomechanical measurements from piezoelectric sensors and the occurrence of rib injuries was assessed. The ROC curve (Figure 9A) illustrates the discriminatory capacity of the biomechanical measurement considered. For ribs, AUC obtained is 0.83, which indicates acceptable predictive performance according to the criteria proposed by (Yoganandan et al., 2016), with an AUC value greater than 0.7 generally considered satisfactory.

The ROC curve analysis also allows us to determine an optimal threshold to diagnostic. For impacts reproduced on the SurHUByx surrogate, the critical value identified is 0.414%. Beyond this threshold, the probability of a rib injury occurring increases significantly. The test identifies 91.7% of true negatives and 80% of true positives. However, this threshold does not directly provide a probabilistic estimate of injury risk.

To obtain a continuous relationship between measured stress and injury probability, the process defined by the standard (ISO, 2013) was applied to construct risk curves. Statistical analyses indicate that the Weibull distribution is the best-fit distribution for the experimental ribs data (Figure 9G). According to this model, a 50% injury probability corresponds to a maximum rib strain of 0.30% (±95% CI: 0.22%–0.39%).

Regarding the lungs, only one injury case could be retained under the experimental conditions (Figure 10A). The sensors integrated into the SurHUByx model, such as those placed in the lungs, are unidirectional and localized. Under these conditions, the validity of a case depends on the location of the impact relative to the sensor. Several injury cases occurred outside this area, above or below the sensor. Including these cases would have introduced additional methodological biases. Thus, it was not possible to generate distribution curves for the lung data. Indeed, the inverse Kaplan-Meier survival curve from the measurements included only one lesion case, preventing the adjustment of Weibull, log-normal or log-logistic distribution models.

Furthermore, the current instrumentation does not allow for the measurement of the maximum depth of penetration during an impact. This limits direct comparisons with the 44 mm limit set by the NIJ standard for assessing the severity of chest trauma.

Secondly, a numerical replication of the impact cases was carried out using the SurHUByx FEM model. This numerical approach allows data to be collected independently of a fixed measurement point, unlike the physical surrogate. It allows the localization of the zones of maximum pressure, deformation and nodal displacement, and thus allows the estimation of the backface intrusion for a direct comparison with the regulatory 44 mm threshold imposed by the current standard (NIJ). The use of the sinking criterion in the different locations is the only criterion which allows the link to be made with the current standard. However, this criterion is based on mortality probabilities in goats, not directly on specific human injuries. For a 4% probability of death, the backface intrusion in goats is 34 mm, corresponding to a depth of 44 mm in Roma Plastilina No. 1 (Clare et al., 1975). Furthermore, how the maximum depth of 44 mm of backface intrusion was translated into response on the human thorax is insufficiently understood (Sarhan et al., 2023). In the absence of alternative normative references to allow analysis of the results, the adoption of the 44 mm threshold serves as a reference.

Simulations showed that, for a 44 mm backface intrusion, the probability of rib fracture is 44.84% (±95% CI: 23.53%–66.53%), and the risk of lung injury is estimated at 52.45% (±95% CI: 18.06%–74.21%) on the SurHUByx FEM model. These results can be compared with the work of Bustamante and Cronin (2024), who also used numerical simulations to replicate impacts on a validated biofidelic human thoracic model (50th percentile male) to evaluate the BFD outcome based on impact location. According to this study, for a 50% risk of rib fracture, the BFD depth is 40.7 mm, a value lower than that observed in the present research (Figure 9H). These results can also be compared with the work of McMahon et al. (2025), who observed a 58.55% risk of liver injury for a 44 mm backface intrusion on PMHS standardized according to a 50th percentile male template. On the other hand, a direct comparison with the studies of Bir et al. (2004) or Bass et al. (2006) is difficult, due to the differences in the variables, impact depth or maximum sternal displacement.

The critical threshold was also determined for these variables. For backface intrusion associated with ribs injuries, the threshold is 47.97 mm. At this level, the test correctly identifies 75% of positive cases, but results in a high proportion of false positives (40%), corresponding to a Youden index of 0.35. These results indicate that the test works, but its accuracy remains quite limited. For backface intrusion associated with lungs injuries, the critical threshold is 38.06 mm. In this case, the test detects all positive cases without generating false negatives. It should be noted, that these values ​​are based on the available data sample, which remains limited in each category, limiting the generalizability of the results.

The other variables, pressure/time for the lungs and strain X for the ribs, studied from the numerical simulations showed better predictive performance. With AUC values ​​and Youden indices higher than with the penetration variables (ribs and lungs). However, these variables cannot be compared with the current NIJ standard (Figures 9, 11F).

The complementarity and independent use of the two approaches is one of the strengths of this study. The experimental approach allows rapid acquisition of global data thanks to physical sensors installed on the structure, allowing rapid access to information during tests. The sensors can detect only specific phenomena and provide a global view of the structure’s behavior. The numerical approach allows a detailed analysis of the structure’s behavior. However, it requires more processing time, as it is based on computer modeling, which involves a preparation, simulation, and results analysis phase. Numerical simulation makes it possible to obtain local measurements on the entire model, in each element of the structure. It allows for precise identification of stress concentration or potential fracture zones. Moreover, the data obtained from the reported BABT cases, combined with the EF model, provide additional insights into injury mechanisms (Sarhan et al., 2023).

This complementarity also allows for a direct comparison of the results obtained. The numerical strain value along the normal to the x-axis of the ribs could be compared with that obtained by the strain gauges. For a 50% risk of rib fracture, the value obtained via the experimental probability curve is 0.30% (±95% CI: 0.22%–0.39%), while that obtained with the numerical model is 0.21% (±95% CI: 0.12%–0.31%). This difference can be explained by the method of extracting the numerical values, which consists of calculating the average between the maximum value and those of the neighbouring nodes.

The construction of risk curves from survival analyses is a widely used method in trauma biomechanics (Yoganandan et al., 2016). These curves are useful because they allow for a continuous representation of risk as a function of an input variable (Hostetler et al., 2021). This study builds on ISO recommendations as well as previous work (Yoganandan et al., 2016) to establish injury risk curves. The advantage is that it uses known and validated data analysis methods. In addition, it makes the study comparable with other work. The approach adopted here aims to estimate risk from predictions of strain, deflection, and pressure compared to observed injuries, with the aim of anticipating future injury probabilities. However, the results presented in this study are specific to the surrogates used and cannot be generalized to other models without further validation. Indeed, the SurHUByx and SurHUByx FEM surrogates were validated by comparison to the experimental corridors of Bir and Viano (2004), which assess the overall biomechanical response of the thorax. Therefore, the individual components of these surrogates do not have specific validation.

The study also considered individual variability in case reports. The physical characteristics of individuals mentioned in the accident reports were incorporated via a BMI-based scaling factor. This method appears to reduce morphological variations and the case exclusion rate. Chest depth was not used for standardization, as suggested by Bir et al. (2004) and Wilhelm (2003), as the data were not available in the reports. Standardized data from US and French ballistic reports were used to estimate the risk of rib fracture in men of average body type. However, BMI-based standardization has its limitations. For example, the mechanical properties of ribs can vary depending on the individual’s height and body type (Holcombe et al., 2017). In this study, one case report (BABTID #051) had to be excluded from the analyses because the individual characteristics were extreme compared to those of a 50th percentile male. Despite standardization of the different values, the data obtained for this case were aberrant.

Finally, collecting real-life cases of ballistic injuries remains difficult. The cases analyzed in this study constitute a valuable database, with each case allowing for the refinement of BABT injury probability estimates. However, the total number of cases remains limited. This methodological weakness prevents reliable modeling of a risk curve for this type of injury. A resampling approach, such as that proposed by DeVogel et al. (2019), could be considered to balance the data classes and improve the robustness of the results.

The perspectives for this research include the expansion of the BABT database to establish injury risk curves for all vital organs. Furthermore, since this study was based exclusively on male subjects corresponding to the 50th percentile, future works need to take into account morphological diversity, particularly that of women, in order to improve the representativeness of predictive models.

## 5 Conclusion

This study demonstrated the value of modeling and predicting injuries due to non-penetrating ballistic impacts using both numerical and physical surrogates (SurHUByx and SurHUByx FEM). The use of the physical and numerical models enabled the collection of global and local data. By reproducing real BABT cases with validated surrogates representing a 50th percentile male thorax, injury risk curves were established for rib fractures and lung injuries. This study highlighted the correlation between measured strain levels and rib fracture probabilities from known BABT injury cases, validating the approach based on integrated sensors. However, the results for lung injuries remain limited. Nonetheless, the case with a lung injury corresponds to the one where the piezoelectric sensor signal was the most important, which indicates a potential interest of this instrumentation to detect this type of injury. Numerical results revealed a 44.84% risk of rib fracture and a 52.45% risk of lung injury at the current 44 mm BFD threshold imposed by the NIJ standard. These results suggest that current ballistic protection criteria may not provide optimal protection against internal injuries. Although the study is limited by number of documented BABT cases available and the uneven distribution of injuries, it provides a robust framework for future injury risk modeling.

## Data Availability

The datasets presented in this article are not readily available because the reports from the French Ministry of Interior are confidential. The data cannot be made public. Requests to access the datasets should be directed to elodie.dancerelbourlon@uphf.fr.
